# Hollow TiO_2_@Co_9_S_8_ Core–Branch Arrays as Bifunctional Electrocatalysts for Efficient Oxygen/Hydrogen Production

**DOI:** 10.1002/advs.201700772

**Published:** 2017-12-19

**Authors:** Shengjue Deng, Yu Zhong, Yinxiang Zeng, Yadong Wang, Xiuli Wang, Xihong Lu, Xinhui Xia, Jiangping Tu

**Affiliations:** ^1^ State Key Laboratory of Silicon Materials Key Laboratory of Advanced Materials and Applications for Batteries of Zhejiang Province Zhejiang University Hangzhou 310027 P. R. China; ^2^ School of Applied Physics and Materials Wuyi University Jiangmen Guangdong 529020 China; ^3^ School of Engineering Nanyang Polytechnic Singapore 569830 Singapore

**Keywords:** arrays, cobalt sulfide, electrochemical water splitting, hydrogen evolution reaction, oxygen evolution reaction

## Abstract

Designing ever more efficient and cost‐effective bifunctional electrocatalysts for oxygen/hydrogen evolution reactions (OER/HER) is greatly vital and challenging. Here, a new type of binder‐free hollow TiO_2_@Co_9_S_8_ core–branch arrays is developed as highly active OER and HER electrocatalysts for stable overall water splitting. Hollow core–branch arrays of TiO_2_@Co_9_S_8_ are readily realized by the rational combination of crosslinked Co_9_S_8_ nanoflakes on TiO_2_ core via a facile and powerful sulfurization strategy. Arising from larger active surface area, richer/shorter transfer channels for ions/electrons, and reinforced structural stability, the as‐obtained TiO_2_@Co_9_S_8_ core–branch arrays show noticeable exceptional electrocatalytic performance, with low overpotentials of 240 and 139 mV at 10 mA cm^−2^ as well as low Tafel slopes of 55 and 65 mV Dec^−1^ for OER and HER in alkaline medium, respectively. Impressively, the electrolysis cell based on the TiO_2_@Co_9_S_8_ arrays as both cathode and anode exhibits a remarkably low water splitting voltage of 1.56 V at 10 mA cm^−2^ and long‐term durability with no decay after 10 d. The versatile fabrication protocol and smart branch‐core design provide a new way to construct other advanced metal sulfides for energy conversion and storage.

Developing green fuel technology is critical for energy security and sustainable development of social economy. Electrochemical water splitting is recognized as a highly potential technology to convert electricity into environment friendly and renewable chemical fuels (hydrogen and oxygen).^1^, ^2^, ^3^ The cathodic hydrogen evolution reaction (HER) and anodic oxygen evolution reaction (OER) depend heavily on the development of cost‐effective high‐performance electrocatalysts.^4^, ^5^ Currently, platinum (Pt)/Pt‐based alloy and iridium/ruthenium oxides (IrO_2_/RuO_2_) are considered as the most promising electrocatalysts for HER and OER, respectively, but their scarcity, high cost, and compromised stability hinder their widespread applications.^6^, ^7^ Additionally, the best working situation for those OER and HER catalysts is often mismatchable since OER preferably takes place in alkaline or neutral solution while HER in acidic medium.^8^, ^9^ This would cause compromised performance for overall water splitting. For instance, the commercial alkaline electrolyzers require high cell voltages (1.8–2.0 V) to drive water splitting,^10^ far ahead of the theoretical value of ≈1.23 V owing to high overpotentials on the sluggish of OER and HER. Therefore, it is highly desirable to explore alternative high‐performance and low‐cost bifunctional OER/HER electrocatalysts for overall water splitting.^11^, ^12^, ^13^, ^14^, ^15^

Over the past decades, great progress has been achieved on the development of non‐noble metal‐based electrocatalysts for both OER and HER. Various nonprecious metal oxides,^9^ sulfides,^16^ selenides,^17^ phosphides, and nitrides^18^, ^19^ have been exploited. Among these electrocatalysts, cobalt sulfide (Co_9_S_8_) is regarded as an attractive electrocatalyst for water splitting due to its high catalytic activity for HER and OER simultaneously, and excellent electrochemical stability. In comparison to bulk Co_9_S_8_, nanostructured Co_9_S_8_ and its composites could afford more active sites and faster transfer rate of ions/electrons during the electrocatalytic reaction, and thus usually exhibiting enhanced HER and OER activities.^20^, ^21^, ^22^, ^23^, ^24^ Currently, several Co_9_S_8_ nanostructures (e.g., nanoparticles^25^ and nanospheres^26^) and their composites with carbons (Co_9_S_8_/reduced graphene oxides (RGO),^22^ Co_9_S_8_/(N, S, P)‐doped carbons,^27^ Co_9_S_8_/Fe_3_O_4_/RGO,^23^ and Co_9_S_8_/MoS_2_/Carbon fibres^25^) have been reported. For example, Co_9_S_8_/N,P‐carbon powder nanocomposites prepared by molten‐salt calcination method at 900 °C exhibited an HER overpotential of 261 mV at 10 mA cm^−2^ in alkaline medium.^20^ N‐Co_9_S_8_/graphene nanocomposites was achieved with an OER Tafel slope of 82.7 mV Dec^−1^ and a overpotential of ≈0.41 V at 10 mA cm^−2^ by a hydrothermal method.^22^ In spite of enhanced electrocatalytic performance to some extent, the water splitting activity of the aforementioned Co_9_S_8_‐based catalysts is still not satisfactory. One hand, the catalytic performance of powder Co_9_S_8_‐based catalysts is greatly undermined because the active sites would be covered or annihilated during the preparation of test electrode with polymer binders.^28^ Moreover, powder‐form materials are prone to detach from the surface of test electrode at large working currents due to the bubble striking effect. This can greatly reduce life span and increase inner resistance. Additionally, numerous undesirable interfaces and extra resistance are inevitably introduced leading to higher overpotentials.^29^ On the other hand, the synthetic condition for the Co_9_S_8_‐base catalysts in these published works is always harsh with high temperature sintering and heavily polluted by using H_2_S or organic precursors with sulfur sources (such as thiourea and trithiocyanuric acid).^30^, ^31^, ^32^ In such a context, green and low‐temperature sulfurization method must be established to fabricate high‐activity binder‐free Co_9_S_8_‐base catalysts to achieve high performance.

In the present work, we report a simple and powerful sulfurization strategy to rationally design hollow TiO_2_@Co_9_S_8_ core–branch arrays for the first time as robust bifunctional electrocatalysts for both OER and HER in alkaline medium. The bind‐free TiO_2_@Co_9_S_8_ core–branch arrays are proven with large porosity/surface area and strong adhesion on the conductive substrates, endowing them with more active cites, faster ions/electrons transport rate and better structural stability. Such unique structural features enable the TiO_2_@Co_9_S_8_ core–branch arrays to deliver remarkably enhanced HER and OER properties compared to pristine Co_9_S_8_ nanowires. Low overpotentials of 240 and 139 mV at 10 mA cm^−2^ as well as small Tafel slopes of 55 and 65 mV Dec^−1^ for OER and HER in alkaline medium are achieved by our TiO_2_@Co_9_S_8_ core–branch electrode, respectively. More importantly, an advanced electrolysis cell with a highly low water splitting voltage of 1.56 V at 10 mA cm^−2^ and excellent durability (no any decay after soaking in the electrolyte for 10 d) is demonstrated when using the TiO_2_@Co_9_S_8_ core–branch arrays as both cathode and anode, outperforming most of the developed electrochemical water splitting cells. Our novel electrode design/fabrication protocol can provide a reference for construction of high‐performance integrated branch‐core arrays for applications in electrocatalysis and energy storage.

The TiO_2_@Co_9_S_8_ hollow core–branch arrays are prepared via a low‐temperature sulfurization on the preformed TiO_2_@Co_2_(OH)_2_CO_3_ core–shell arrays (Figure S1, Supporting Information). First, the Co_2_(OH)_2_CO_3_ nanowire arrays on the nickel foam substrate are prepared by a simple hydrothermal synthesis. Apparently, the sample shows red color and homogeneous Co_2_(OH)_2_CO_3_ nanowires of 80–100 nm are grown quasi‐vertically onto the substrate (Figure S2a–c, Supporting Information). These nanowires have a smooth surface and grow independently with noninterference, leaving a 3D porous structure. Transmission electron microscope (TEM) images and selected area electron diffraction pattern demonstrate the smooth texture and single crystalline characteristics of the Co_2_(OH)_2_CO_3_ nanowire (Figure S2d–f, Supporting Information). The existence of Co_2_(OH)_2_CO_3_ (Joint Committee of Powder Diffraction Standards (JCPDS) 48‐0083) is supported by X‐ray diffraction (XRD) and Raman analysis (Figure S2g–h, Supporting Information). Then, a thin atomic layer deposition (ALD)‐TiO_2_ layer of ≈10 nm is deposited on the Co(OH)_2_CO_3_ nanowires to form TiO_2_@Co(OH)_2_CO_3_ core–shell nanowire arrays (Figure S3a–e, Supporting Information). The color of sample turns into pale pink color (inset in Figure S3a, Supporting Information). TEM and high‐resolution transmission electron microscopy (HRTEM) images in Figure S3d–f in the Supporting Information clearly verify the core–shell structure and amorphous nature of the TiO_2_ layer of ≈10 nm. The coexistence of Co_2_(OH)_2_CO_3_ and TiO_2_ is further verified by XRD and Raman analysis (Figure S3g–h, Supporting Information). Finally, the hollow TiO_2_@Co_9_S_8_ core–branch arrays are obtained after treating the TiO_2_@Co(OH)_2_CO_3_ nanowires in 0.1 m Na_2_S solution at 90 °C for 9 h. After sulfurization, the color of sample turns to black due to the formation of Co_9_S_8_ (inset in **Figure** **1**a and Figure S4, Supporting Information). Interestingly, the previous dense core–shell structure of TiO_2_@Co(OH)_2_CO_3_ disappears and perfect 3D hollow core–branch arrays architecture is successfully formed (Figure 1b,c and Figure S4a–c, Supporting Information). The internal TiO_2_ nanotube core is uniformly decorated by the shell consisting of crosslinked Co_9_S_8_ nanoflakes with thicknesses of 10–15 nm. The diameter for the TiO_2_@Co_9_S_8_ core–branch structure is about 400–450 nm. The diffraction rings of (400), (331) in SAED pattern (inset in Figure 1c) and the measured layer spacing of about 0.30 nm corresponding to crystal planes of (311) indicate the existence of Co_9_S_8_ phase (Figure 1d). Energy dispersive X‐ray (EDS) mapping images (Figure 1e–f) also indicate the presence and homogeneous distribution of Co, S, Ti, and O elements in TiO_2_@Co_9_S_8_ arrays samples, which further prove the hollow TiO_2_ nanotube core and branch Co_9_S_8_ shell. It is noteworthy that the TiO_2_ layer is vital to the formation of hollow TiO_2_@Co_9_S_8_ core–branch architecture. Only common Co_9_S_8_ nanowire arrays are formed without the presence of TiO_2_ layer (Figure S5, Supporting Information). Furthermore, our present synthetic strategy is also versatile and powerful for growth on different substrates (e.g., carbon cloth, Figure S6, Supporting Information). To reveal this sulfurization mechanism, it is inferred that there are two sulfurization pathways for the growth of Co_9_S_8_. In the presence of TiO_2_ layer, the plausible reaction mechanism, most likely, is associated with the “oriented attachment” and “induced self‐assembly” effects (Figure S7, Supporting Information). It is known that the Na_2_S solution hydrolyzes and produces acid species (e.g., HS—, H_2_S, etc.), which can react with the basic Co_2_(OH)_2_CO_3_. The reactants with S sources are prone to preconcentrate along the outer surface of the TiO_2_ layer. In the meantime, the dissolved Co cations transport outward to meet the S sources to form Co_9_S_8_ crystals leading to the disappearance of Co_2_(OH)_2_CO_3_ nanowire. Here the TiO_2_ layer acts as the backbone for heterogeneous nucleation and guides the preferential growth of Co_9_S_8_. This process is believed to be involved with spontaneous “oriented attachment” and “induced self‐assembly” of adjacent Co_9_S_8_ particles when supersaturated solution with considerable Co_9_S_8_ crystals is formed. The Co_9_S_8_ crystals are attached to the surface of TiO_2_ layer to generate active nucleation centers, which would minimize the interfacial energy barrier for the subsequent growth of Co_9_S_8_. Finally, these Co_9_S_8_ crystals self‐assemble with each other resulting in the formation of TiO_2_@Co_9_S_8_ hollow core–branch arrays. On the other hand, without the TiO_2_ layer, the direct conversion would take place and produce common Co_9_S_8_ nanowire arrays.

**Figure 1:**
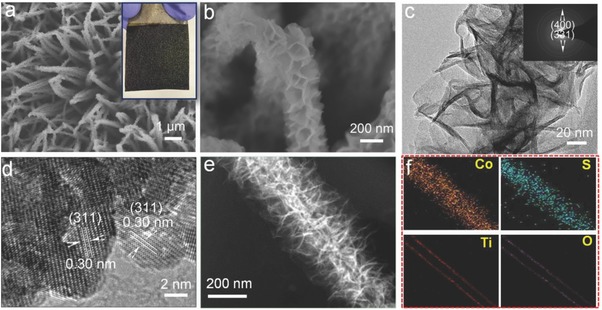
a,b) Scanning electron microscopy (SEM) images (optical photograph in inset); c) TEM image (selected area electron diffraction (SAED) pattern in inset); d) HRTEM image; e) high‐angle annular dark‐field scanning TEM (STEM) image; and f) EDS elemental mapping images of Co, S, Ti, and O of the TiO_2_@Co_9_S_8_ hollow core–branch arrays.

To further highlight the benefits of core–branch hollow arrays, the specific surface area was measured by Brunner−Emmet−Teller (BET) analysis (Figure S8, Supporting Information). The pristine Co_9_S_8_ nanowire arrays and TiO_2_@Co_9_S_8_ core–branch arrays grown on the nickel foam substrate exhibit a specific surface area of 1.4 and 4.0 m^2^ g^−1^, respectively, indicating that the design of hollow core–branch structure can greatly increase the surface area. It is noteworthy that the nickel foam substrate accounts for about 88% and 83% in the weight of nickel foam supported TiO_2_@Co_9_S_8_ and Co_9_S_8_ nanowire samples, respectively. As a result, the specific surface area of the individual TiO_2_@Co_9_S_8_ is estimated to be ≈33.4 m^2^ g^−1^ excluding the nickel foam, far ahead of the Co_9_S_8_ nanowire samples (≈8 m^2^ g^−1^). It suggests that the core–branch structure is favorable for providing more active area/sites exposed and improve the utilization of active Co_9_S_8_ catalysts.

X‐ray photoelectron spectroscopy (XPS) and Raman tests were performed to further determine the phase and composition. For the TiO_2_@Co_9_S_8_ arrays, the XPS survey spectrum verifies the presence of Co, S, Ti, and O elements (**Figure** **2**a), consistent with the analysis of EDS mapping above. Only Co, S, and O element exist in the Co_9_S_8_ nanowire arrays. The O element in Co_9_S_8_ nanowire sample may be from OH^−^.^33^ Figure 2b shows the high‐resolution S 2p spectra of both samples. Two core levels (S 2p3/2 and S 2p1/2) are located at 161.3 and 163.1 eV, respectively, which match well with the electronic states of Co_9_S_8_. The high‐resolution Co 2p spectra of both samples possess typical core levels of Co 2p1/2 (796.5 eV) and Co 2p3/2 (780.7 eV) and two satellite peaks (Figure 2c).^33^, ^34^, ^35^ Meanwhile, the presence of TiO_2_ in the TiO_2_@Co_9_S_8_ arrays is also supported by Ti 2p and O1s spectra (Figure S9, Supporting Information). The above results are strongly supported by Raman analysis (Figure 2d). Five characteristic peaks of Co_9_S_8_ phase (218, 253, 316, 373, and 685 cm^−1^) are found in both samples. Moreover, the TiO_2_@Co_9_S_8_ sample has a new peak at 150 cm^−1^, revealing the existence of TiO_2_.^36^ All these results mutually support that TiO_2_@Co_9_S_8_ and Co_9_S_8_ nanowire arrays are successfully fabricated via our facile sulfurization method.

**Figure 2:**
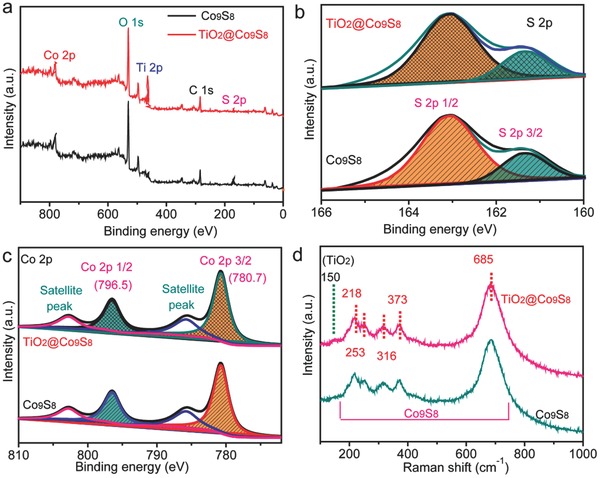
XPS and Raman characterizations of TiO_2_@Co_9_S_8_ and Co_9_S_8_ arrays: a) XPS survey spectra; b) core‐level S 2p XPS spectra; c) core‐level Co 2p XPS spectra; and d) Raman spectra of the Co_9_S_8_ and TiO_2_@Co_9_S_8_ arrays.

The electrochemical application of TiO_2_@Co_9_S_8_ arrays as electrocatalysts for water splitting is thoroughly characterized. First, the electrochemical OER activities of the samples were investigated by using a simple three‐electrode system in 1 m KOH solution. In our experiment, four different electrodes (Co_2_(OH)_2_CO_3_ nanowire arrays, TiO_2_@Co_2_(OH)_2_CO_3_ core–shell arrays, Co_9_S_8_ nanowire arrays, and TiO_2_@Co_9_S_8_ core–branch arrays) are selected for OER comparison. **Figure** **3**a presents the linear sweep voltammetry (LSV) curves of these four different electrodes. Significantly, the TiO_2_@Co_9_S_8_ electrode exhibits the best OER catalytic performance with the lowest overpotential (240 mV at the current density of 10 mA cm^−2^), superior to the Co_9_S_8_ (276 mV), Co_2_(OH)_2_CO_3_ (330 mV), and TiO_2_@Co_2_(OH)_2_CO_3_ (350 mV) electrodes. The present overpotential is also substantially lower than recently reported Co_9_S_8_‐based catalysts at the same current density, such as Co_9_S_8_@N and S codoped porous carbon tube (310 mV),^27^ hollow Co_9_S_8_ microplates (273 mV),^26^ and Fe_3_O_4_@Co_9_S_8_/rGO (340 mV).^27^ This indicates the construction of hollow core–branch architecture is favorable for the reinforcement of OER, further proven by the Tafel slope analysis (Figure 3b). The TiO_2_@Co_9_S_8_ electrode exhibits the lowest of Tafel slope of 55 mV dec^−1^, much better than other counterparts, suggesting its fastest OER process (Table S1, Supporting Information). To deepen the understanding of the enhanced OER activity, the effective electrochemical active surface area (ECSA) of all samples was estimated by testing the double‐layer capacitance (DLC) according to the cyclic voltammetry (CV) results at different scan rates (Figure S10, Supporting Information).^37^ The obtained current density is plotted as a function with scan rates in Figure 3c. The ECSA value is linearly proportional to the DLC value, equaling to the half of the slope value. Remarkably, the highest capacitance up to 48 mF cm^−2^ is achieved by the TiO_2_@Co_9_S_8_ electrode, demonstrating its largest ECSA, much larger than the Co_2_(OH)_2_CO_3_ (28 mF cm^−2^), TiO_2_@Co_2_(OH)_2_CO_3_ (11 mF cm^−2^), and Co_9_S_8_ (34 mF cm^−2^) electrodes, indicating the markedly improved active areas for the TiO_2_@Co_9_S_8_ electrode. Furthermore, as presented in Figure 3d, the smallest circle diameter of the TiO_2_@Co_9_S_8_ electrode shows that it possesses the lowest charge transfer resistance (*R*_ct_), revealing that the TiO_2_@Co_9_S_8_ electrode has faster electrocatalytic reaction kinetics. The unique design of hollow core–branch arrays provides positive effects in the enhancement of OER performance. (1) Direct growth of TiO_2_@Co_9_S_8_ arrays on conductive substrates avoids the use of insulated polymer binders and annihilation of active sites. (2) The crosslinked Co_9_S_8_ nanoflakes with large specific surface area increase accessible area between active materials and electrolyte and provide more active sites. Moreover, the hollow core can serve as buffer “electrolyte reservoirs” to accelerate the transport of ions leading to faster catalytic reactions. In addition, the porous and hollow structure is beneficial for overflow of O_2_ and will not block the active sites during water splitting processes. Noticeably, the TiO_2_@Co_9_S_8_ core–branch electrode also possesses excellent long‐term OER durability. As shown in Figure 3e, the TiO_2_@Co_9_S_8_ electrode retains higher activity and more stable life span than the Co_9_S_8_ nanowire electrode at different current densities ranging from 10 to 50 mA cm^−2^.

**Figure 3:**
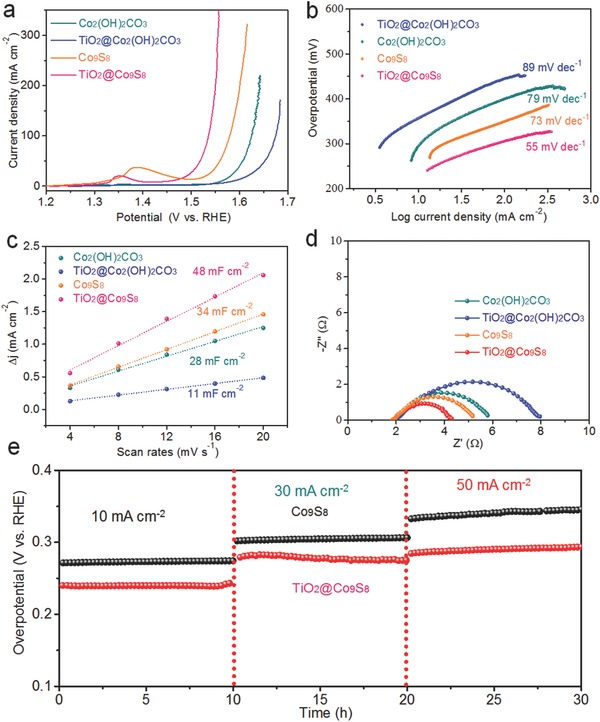
OER performances: a) LSV curves at 5 mV s^−1^; b) Tafel plots; c) the ratio of current density with various scan rates; and d) Nyquist plots of Co_2_(OH)_2_CO_3_, TiO_2_@Co_2_(OH)_2_CO_3_, Co_9_S_8_ and TiO_2_@Co_9_S_8_ electrodes; e) electrochemical stability of the Co_9_S_8_ and TiO_2_@Co_9_S_8_ electrodes at different current densities.

Apart from excellent OER activity, the obtained TiO_2_@Co_9_S_8_ electrode also exhibits outstanding HER catalytic performance in alkaline solution. As shown in **Figure** **4**a, the TiO_2_@Co_9_S_8_ electrode presents a remarkably low overpotential of 139 mV at 10 mA cm^−2^, superior to the Co_9_S_8_ (222 mV), Co_2_(OH)_2_CO_3_ (197 mV), and TiO_2_@Co_2_(OH)_2_CO_3_ (226 mV) electrodes. Moreover, the TiO_2_@Co_9_S_8_ electrode displays the lowest Tafel slopes (65 mV Dec^−1^), better than the Co_2_(OH)_2_CO_3_ (102 mV Dec^−1^), TiO_2_@Co_2_(OH)_2_CO_3_ (126 mV Dec^−1^), Co_9_S_8_ electrodes (85 mV Dec^−1^) (Figure 4b) as well as other electrocatalysts (Table S1, Supporting Information). Also, long‐term HER durability is demonstrated for the TiO_2_@Co_9_S_8_ electrode at different current densities (Figure 4c). It is justified that our designed hollow core–branch array architecture can promote the HER activity of Co_9_S_8_.

**Figure 4:**
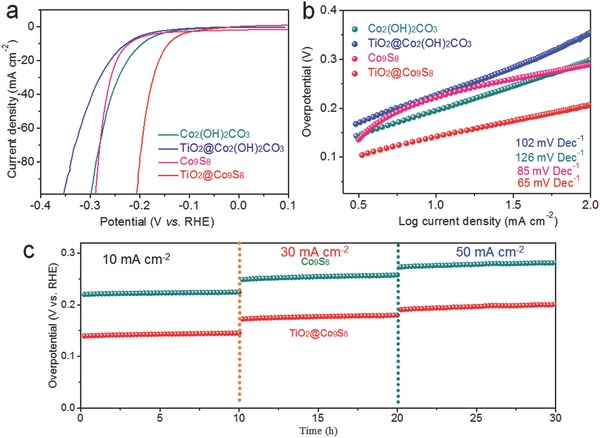
HER performances: a) LSV curves at 5 mV s^−1^; b) Tafel plots of Co_2_(OH)_2_CO_3_, TiO_2_@Co_2_(OH)_2_CO_3_, Co_9_S_8_, and TiO_2_@Co_9_S_8_ electrodes; and c) electrochemical stability of the Co_9_S_8_ and TiO_2_@Co_9_S_8_ electrodes at different current densities and times.

Due to the prominent activities for both OER and HER, the TiO_2_@Co_9_S_8_ electrode could be utilized as an efficient bifunctional electrocatalyst for overall water splitting in alkaline medium. **Figure** **5**a shows the overall water splitting activity of two‐electrode system with the TiO_2_@Co_9_S_8_ electrocatalysts as both cathode and anode in 1 m KOH solution (denoted as TiO_2_@Co_9_S_8_ || TiO_2_@Co_9_S_8_). Impressively, a significantly low cell voltage of 1.56 V is obtained at the current density of 10 mA cm^−2^ (Figure 5a), substantially lower than the Co_9_S_8_ || Co_9_S_8_ catalyzer cell (1.71 V) and other reported bifunctional electrocatalysts,^6^, ^16^, ^27^, ^38^, ^39^, ^40^, ^41^, ^42^, ^43^, ^44^, ^45^, ^46^ such as Co_3_O_4_ (1.63 V), NiCo_2_O_4_ (1.72 V), CoO (1.63 V), NiP (1.63 V), Co_9_S_8_ (1.6 V) (Figure 5b), and even close to the Pt/C || IrO_2_ (1.54 V) catalyzer cell.^44^ Figure 5c compares the chronopotentiometry curves of the TiO_2_@Co_9_S_8_ || TiO_2_@Co_9_S_8_ and Co_9_S_8_ || Co_9_S_8_ catalyzer cells collected at 10 mA cm^−2^. The TiO_2_@Co_9_S_8_ || TiO_2_@Co_9_S_8_ catalyzer cell shows higher activity with lower overpotential for overall water splitting, demonstrating its long‐term durability with no decay after 30 h. In addition, continuous hydrogen and oxygen bubbles could be noticed on the anode and cathode during the stability test, respectively (inset in Figure 5c). SEM and TEM images reveal that the uniform hollow core–branch arrays structure is still well preserved after 30 h (Figure S11, Supporting Information), demonstrating the excellent structural stability of the TiO_2_@Co_9_S_8_ electrode. This is mainly due to the high mechanical strength of TiO_2_ core and good adhesion between TiO_2_ and Co_9_S_8_ nanoflakes. In addition, in order to meet practical application, the LSV performance is performed after soaking the TiO_2_@Co_9_S_8_ electrode in the electrolyte for 10 d (when current is not flowing). Impressively, the whole performance is very stable and water splitting voltage does not show any decay. All the above results indicate that this core–branch hollow structure would remarkably improve electrochemical activities for OER/HER. This makes the TiO_2_@Co_9_S_8_ hollow core–branch arrays promising catalysts for practical application in alkaline water splitting.

**Figure 5:**
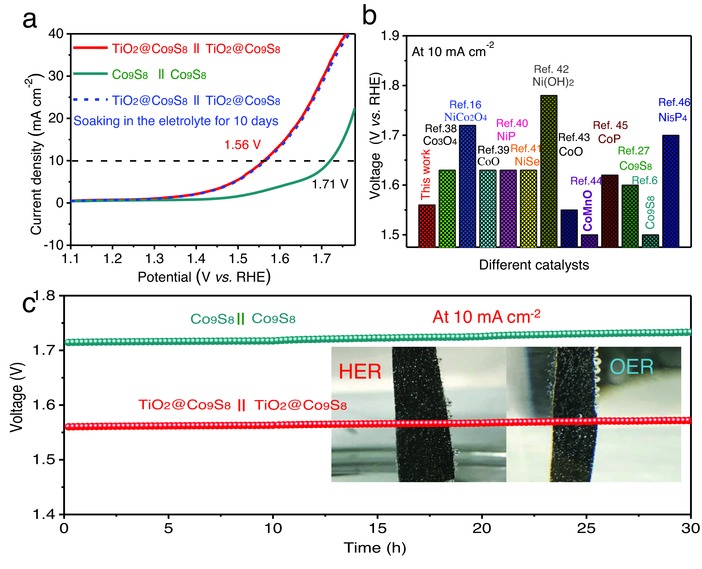
Overall water splitting performance of electrolysis cells: TiO_2_@Co_9_S_8_|| TiO_2_@Co_9_S_8_ and Co_9_S_8_||Co_9_S_8_. a) LSV curves; b) comparison of overall water splitting performance between TiO_2_@Co_9_S_8_ || TiO_2_@Co_9_S_8_ and other electrocatalysts in the literature, and c) electrochemical stability at 10 mA cm^−2^.

In summary, we have demonstrated a facile and high‐efficiency sulfurization approach to realize the rational synthesis of hollow TiO_2_@Co_9_S_8_ core–branch arrays as robust bifunctional electrocatalysts for both OER and HER in alkaline. Crosslinked Co_9_S_8_ nanoflakes are uniformly assembled on the hollow TiO_2_ core forming free‐standing arrays. Meanwhile, the proposed synthetic method is versatile and applicable to different conductive substrates and core–branch morphology. Due to enhanced surface area and porosity, and binder‐free adhesion with the conductive substrate, the designed TiO_2_@Co_9_S_8_ arrays are utilized as bifunctional catalysts for OER/HER and proven with excellent performances with low Tafel slopes and overpotentials and superior cycling stability. Moreover, a low voltage (≈1.56 V) for overall water splitting is achieved in the TiO_2_@Co_9_S_8_ || TiO_2_@Co_9_S_8_ catalyzer cell, superior to other metal sulfides/oxides in the literature. Our work opens a new door to construct advanced electrocatalysts based on novel hollow core–branch array architecture.

## Experimental Section

*Preparation of TiO_2_@Co_9_S_8_ Core–Branch Arrays*: Uniform Co_2_(OH)_2_CO_3_ nanowires arrays were prepared by a simple hydrothermal method. First, 0.75 g Co(NO_3_)_2_, 0.25 g NH_4_F, and 0.75 g CO(NH_2_)_2_ were dissolved in 75 mL deionized water to form hydrothermal solution. Then the above solution was transferred into a Teflon‐linked steel autoclave, which was kept at 120 °C for 6 h. After naturally cooling, the Co_2_(OH)_2_CO_3_ nanowires arrays were rinsed by deionized water. Then, the above Co_2_(OH)_2_CO_3_ nanowire arrays were coated with a layer of TiO_2_ (≈10 nm) by ALD (Beneq TFS 200) with TiCl_4_ and H_2_O as the Ti and O precursors at 120 °C for 140 cycles. Then, in a typical sulfurization process, the above TiO_2_@Co(OH)_2_CO_3_ nanowires arrays were immersed into 0.1 m Na_2_S solution and kept at 90 °C for 9 h. After naturally cooling, the obtained TiO_2_@Co_9_S_8_ core–branch arrays were rinsed by deionized water. For comparison, the Co_9_S_8_ nanowires arrays were prepared by a direct sulfurization procedure for Co_2_(OH)_2_CO_3_ nanowires arrays as the same sulfurization parameters above.

*Material Characterization*: Morphologies and microstructures of all samples were characterized by using a field emission scanning electron microscope (Hitachi SU8010) and the TEM (JEOL 2100F). Specific surface areas distributions were characterized by using Porosity Instruments (BET, JW‐BK112). The crystal structure of all samples was characterized by using XRD reactor with Cu Ka radiation (RigakuD/Max‐2550). Raman spectra were obtained by using RenishawinVia Raman microscopy under 514 nm laser excitation. X‐ray photoelectron spectroscopy was tested by using an Al Ka source with an ESCALAB_250Xi X‐ray photoelectron spectrometer.

*Electrochemical Characterizations*: OER and HER performances of all samples were performed by using an electrochemical workstation (CH Instrument 660D) with a standard three‐electrode setup at room temperature, where carbon rod (*D* = 8 mm) and saturated calomel electrode (SCE) were used as the counter electrode and reference electrode, respectively. The as‐prepared samples were used as the working electrode. The electrolyte of electrochemical tests was 1 m KOH solution. All potentials in this manuscript are referred to the reversible hydrogen electrode (RHE). The conversion of potential between E(RHE) and E(SCE) obeys the following equation: E(RHE) = E(SCE) + 1.0714 V. All samples were first performed the CV test at 50 mV s^−1^ to stabilize the current. The LSV tests were performed at a scan rate of 5 mV s^−1^. The Tafel plots were derived from LSV curves with a scan rate of 1 mV s^−1^. The electrochemical impedance spectroscopy were conducted at the polarization voltage corresponding to current density of 10 mA cm^−2^, in a frequency range from 100 kHz to 50 mHz with an AC amplitude of 10 mV. The stability test was carried out at different constant current densities (10, 30, and 50 mA cm^−2^) for 10 h each. All these results were obtained by iR‐compensation. Overall water splitting was performed in a two‐electrode catalyzer for 30 h at 10 mA cm^−2^, where two TiO_2_@Co_9_S_8_ electrodes with the same geometric area were used as the catalysts for OER and HER, respectively.

## Conflict of Interest

The authors declare no conflict of interest.

## Supporting information

Supplementary
