# Supplementary material for: Hollow TiO2@Co9S8 Core–Branch Arrays as Bifunctional Electrocatalysts for Efficient Oxygen/Hydrogen Production
Source: Adv Sci (Weinh). 2017 Dec 19;5(3):1700772. doi: 10.1002/advs.201700772 (PMC5867071; doi:10.1002/advs.201700772)
Supplement: Supplementary file 1 — Supplementary [file ADVS-5-1700772-s001.pdf]

Copyright WILEY-VCH Verlag GmbH & Co. KGaA, 69469 Weinheim, Germany, 2017.

ADVANCED SCIENCE
Open Access

Supporting Information

for *Adv. Sci.,* DOI: 10.1002/advs.201700772

Hollow TiO_2@Co_9S_8 Core–Branch Arrays as Bifunctional Electrocatalysts for Efficient Oxygen/Hydrogen Production

Shengjue Deng, Yu Zhong, Yinxiang Zeng, Yadong Wang, Xiuli Wang, Xihong Lu, Xinhui Xia,* and Jiangping Tu*

Copyright WILEY-VCH Verlag GmbH & Co. KGaA, 69469 Weinheim, Germany, 2017.

Supporting Information

Hollow $TiO_2@Co_9S_8$ Core-Branch Arrays as Bifunctional Electrocatalysts for Efficient Oxygen/Hydrogen Production

Shengjue Deng[1], Yu Zhong[1], Yinxiang Zeng[2], Yadong Wang[3], Xiuli Wang[1], Xihong Lu[2], Xinhui Xia[1]*, Jiangping Tu[1]*

[1] State Key Laboratory of Silicon Materials, Key Laboratory of Advanced Materials and Applications for Batteries of Zhejiang Province, and Department of Materials Science and Engineering, Zhejiang University, Hangzhou 310027, P. R. China.

[2] MOE of the Key Laboratory of Bioinorganic and Synthetic Chemistry, KLGHEI of Environment and Energy Chemistry, School of Chemistry, Sun Yat-Sen University, Guangzhou 510275, China.

[3] School of Engineering, Nanyang Polytechnic, 569830, Singapore

Correspondence and requests for materials should be addressed to X. Xia (email: helloxxh@zju.edu.cn) and X. Lu (email: luxh6@mail.sysu.edu.cn)

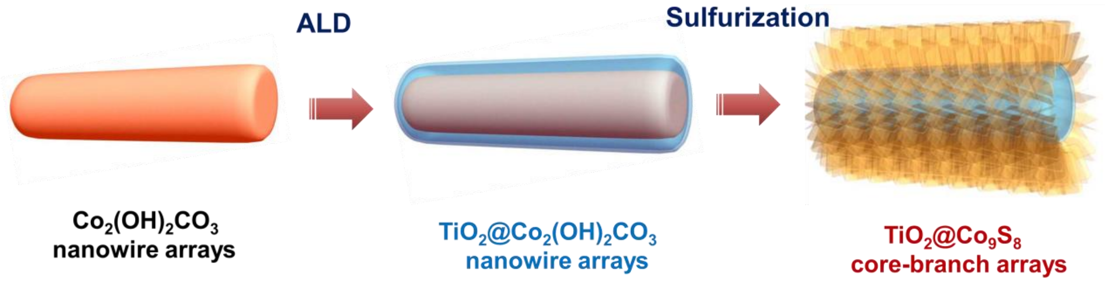

Figure S1. Growth schematics of $TiO_2@Co_9S_8$ hollow core-branch arrays.

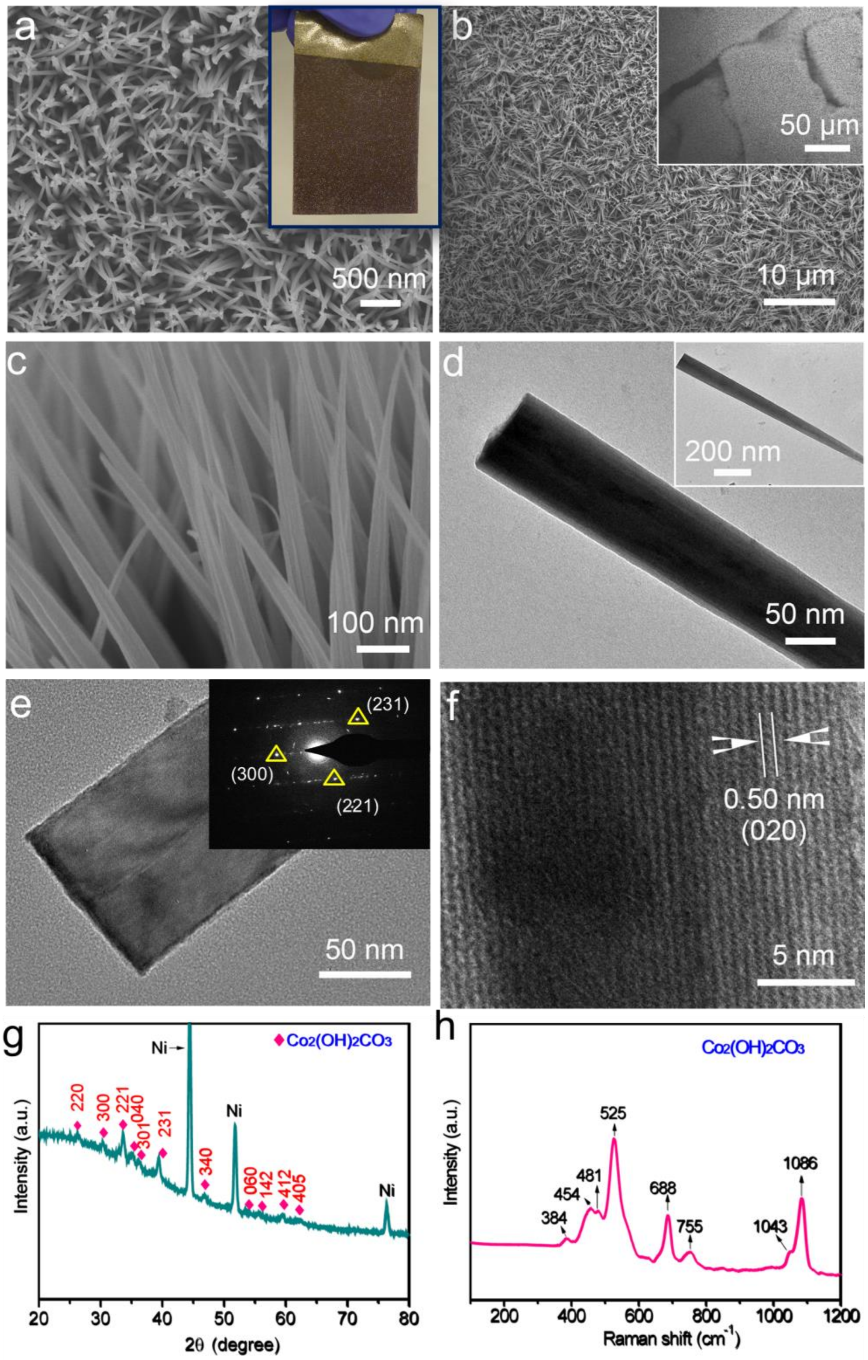

Figure S2. Morphology and microstructure characterizations of $Co_2(OH)_2CO_3$ nanowires arrays: (a-c) SEM images (photo of sample in inset); (d-f) TEM-HRTEM images (SAED pattern in inset); (g) XRD pattern; (h) Raman spectrum.

Except for the peaks of nickel foam substrate, the left diffraction peaks are indexed well with the crystal planes of $Co_2(OH)_2CO_3$ (JCPDS 48-0083) (**Figure S2**g), indicating the formation of high crystallinity of $Co_2(OH)_2CO_3$. Its Raman spectrum (**Figure S2**h) shows eight typical peaks of $Co_2(OH)_2CO_3$ in the region of 200-1100 cm^{-1}.

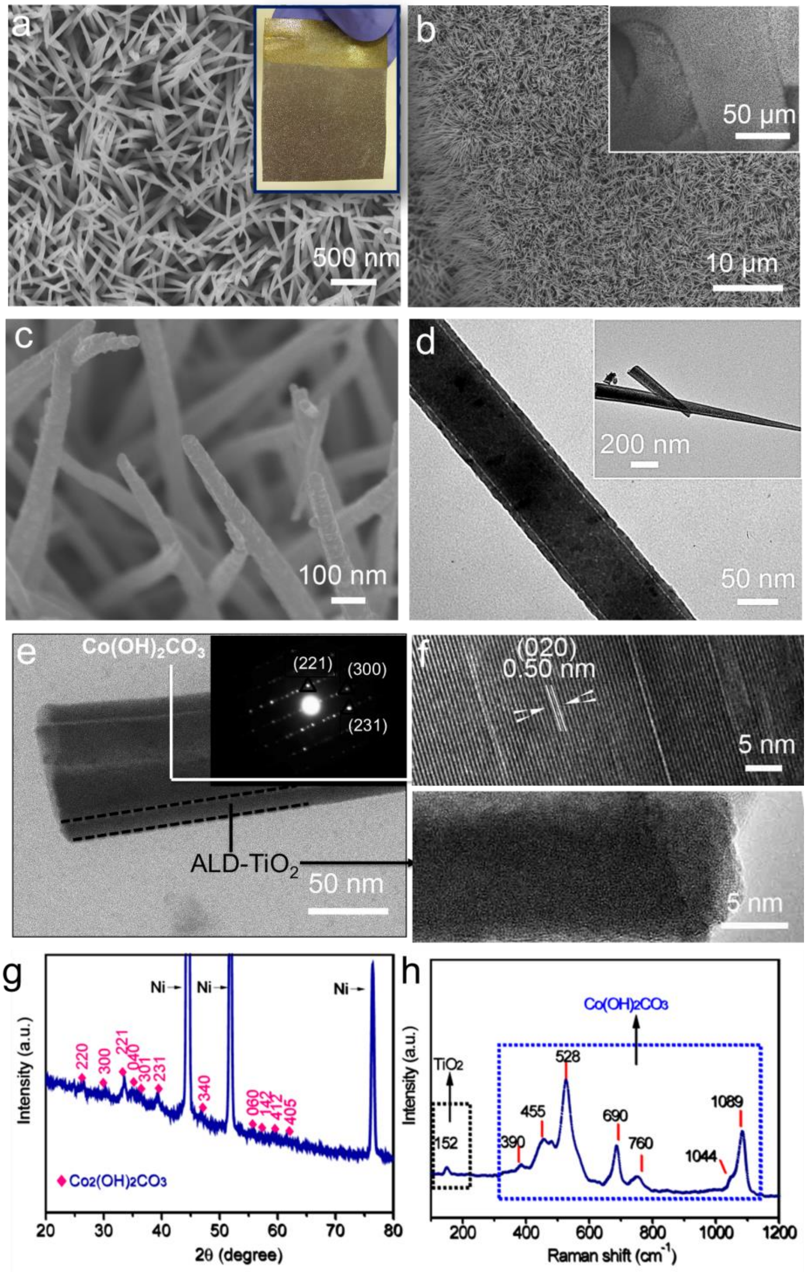

Figure S3. Morphology and microstructure characterizations of TiO_2@$Co(OH)_2CO_3$ core-shell arrays: (a-c) SEM images (photo of sample in inset); (d-f) TEM-HRTEM images (SAED pattern in inset); (g) XRD pattern; (h) Raman spectrum.

Only diffraction peaks of $Co(OH)_2CO_3$ (JCPDS 48-0083) are noticed and no peaks of TiO_2 are detected in the XRD pattern (**Figure S3**g), indicating the amorphous nature of ALD-TiO_2. Additionally, the co-existence of $Co(OH)_2CO_3$ and TiO_2 is verified in the Raman spectrum (**Figure S3**h). In addition to the Raman peaks of $Co_2(OH)_2CO_3$ (Figure S2h), a new characteristic peak of TiO_2 at 150 cm^{-1} is noted.

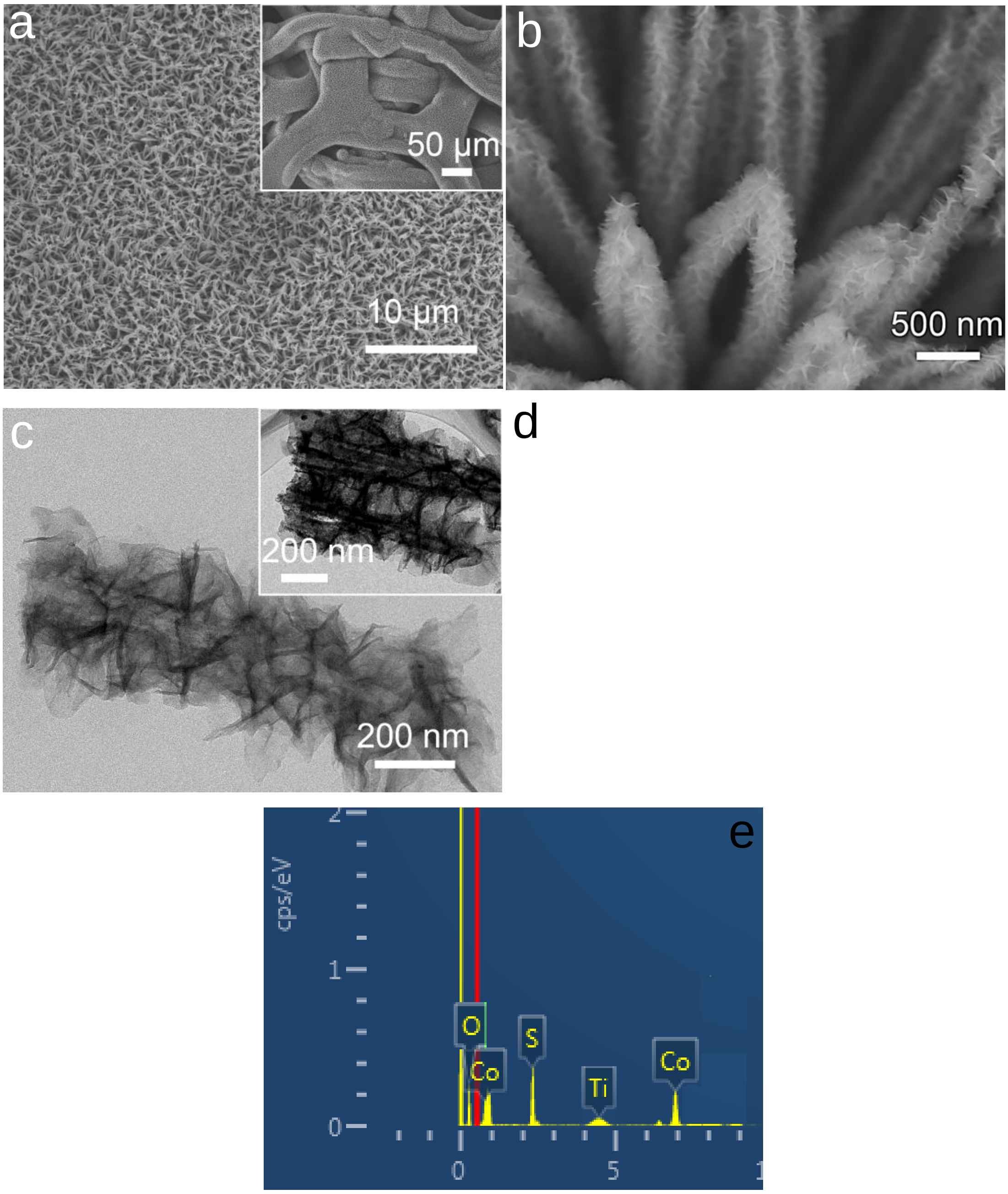

Figure S4. SEM-TEM images of $TiO_2@Co_9S_8$ hollow core-branch arrays on the nickel foam substrate: (a, b) SEM images; (c) TEM image (low-magnification TEM image in inset); and (d) XRD pattern; (e) EDS spectrum of $TiO_2@Co_9S_8$ hollow core-branch arrays in Figure S4c.

The diffraction peaks (311), (22 2), (331) and (531) in XRD pattern are indexed well with the crystal planes of Co_9S_8 phase (JCPDS 65-6801), indicating the successful synthesis of

$TiO_2@Co_9S_8$ arrays on the nickel foam (**Figure S4**d). Also, the above results are supported by the EDS spectrum.

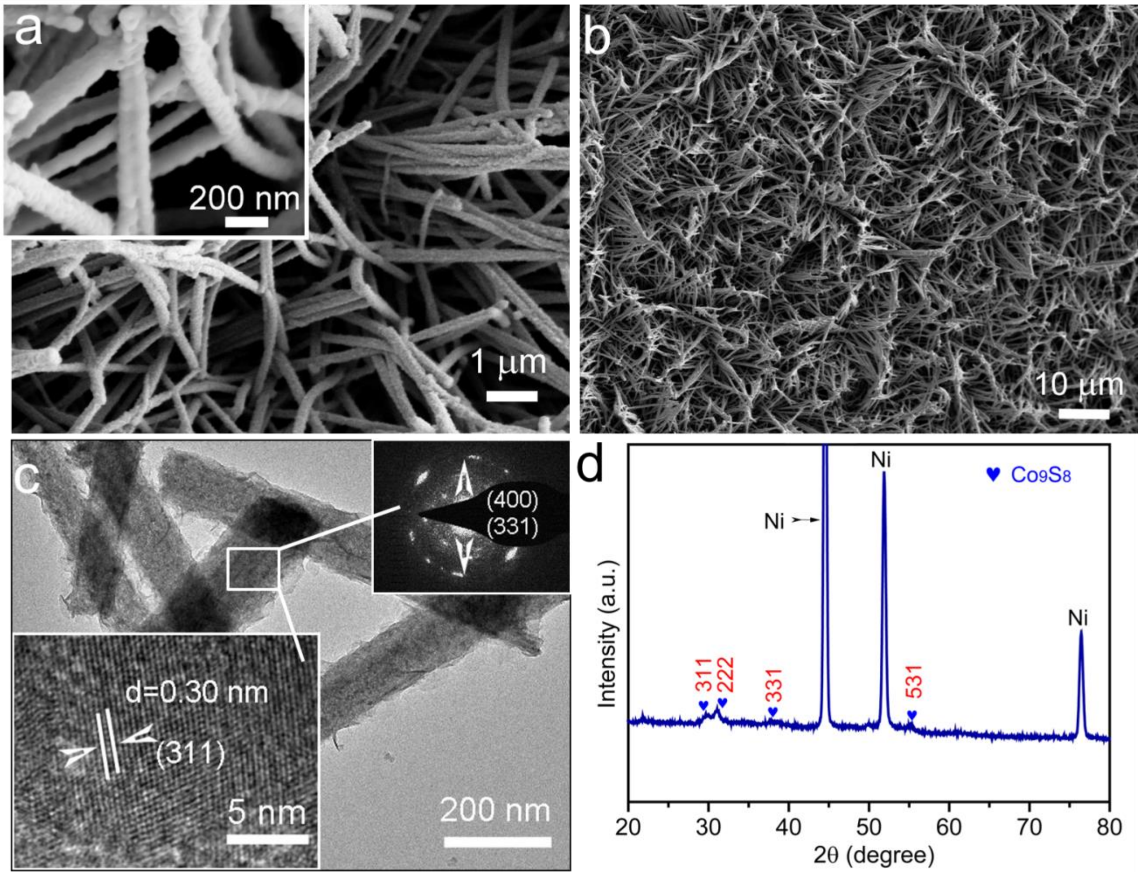

Figure S5. SEM-TEM images of Co_9S_8 nanowires arrays: (a-b) SEM images (inset: high-magnification SEM image); (c) TEM-HRTEM images (SAED pattern and HRTEM image in inset); (d) XRD pattern.

SEM images (**Figure S5**a-b) indicate the uniform distribution of Co_9S_8 nanowires on the nickel foam. TEM-HRTEM and SAED images (**Figure S5**c) reveal the formation of regular Co_9S_8 nanowires. The bright diffraction rings of (400) and (331) demonstrate the existence of high-crystalline Co_9S_8 phase (JCPDS 65-6801). And HRTEM image (inset in **Figure S5**c) exhibits the layer spacing of about 0.30 nm, which matches well with the (311) planes of Co_9S_8 phase, supported by the XRD pattern (JCPDS 65-6801).

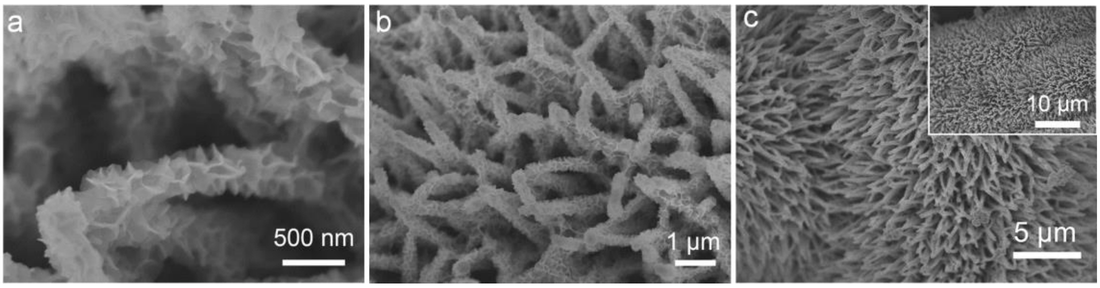

Figure S6. SEM images of TiO_2@Co_9S_8 hollow core-branch arrays grown on the carbon cloth substrate.

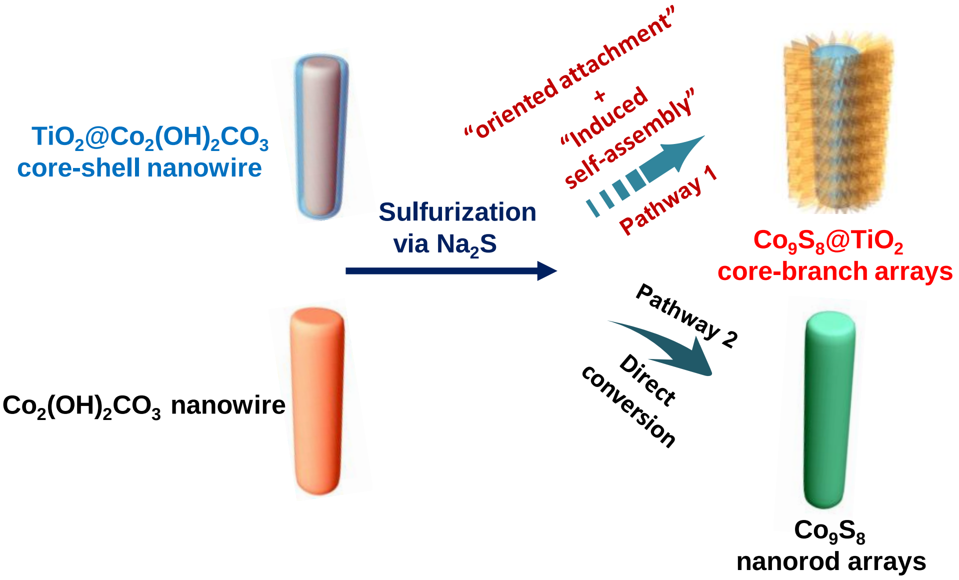

Figure S7. Schematic illustration of the synthesis of Co_9S_8@TiO_2 branch-core and Co9S8 nanowires arrays.

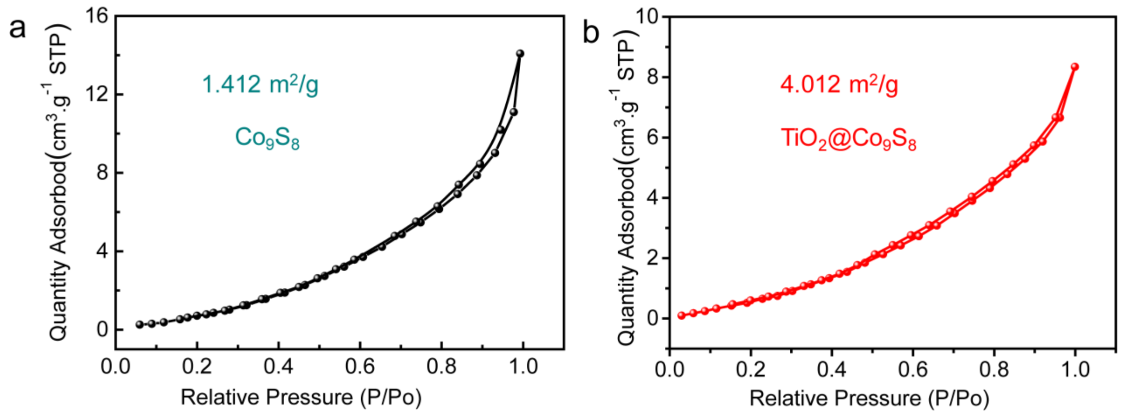

Figure S8. BET measurements: nitrogen adsorption-desorption isotherm curves: (a) Co_9S_8 nanowire arrays and (b) TiO_2@Co_9S_8 hollow core-branch arrays.

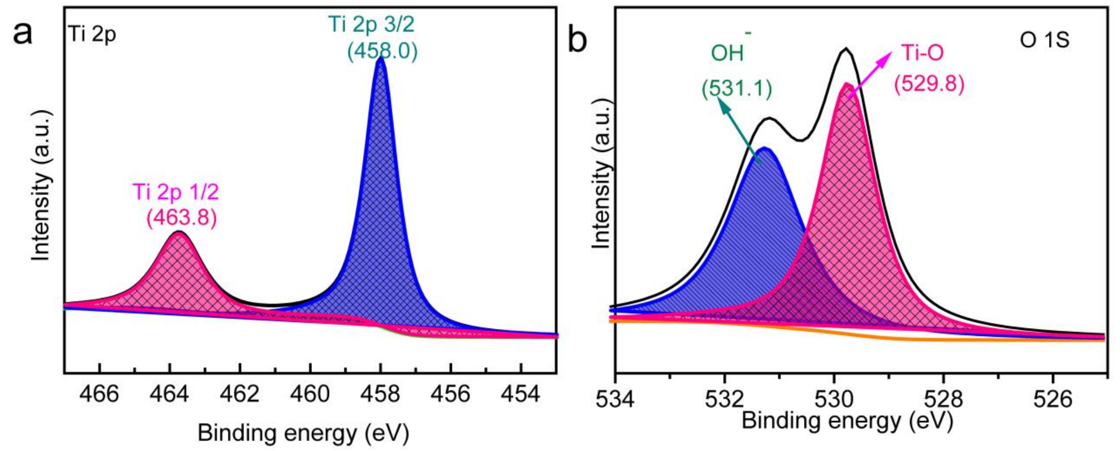

Figure S9. XPS spectra of O 1S and Ti 2p of TiO_2@Co_9S_8 arrays.

Two core levels Ti 2p1/2 (463.8 eV) and Ti 2p3/2 (458.0 eV) characteristic of TiO_2 are detected (**Figure S9**a).[1] Accordingly, Ti-O bond (529.8 eV) is noticed, while the peak at 531.1 eV belongs to OH^- (**Figure S9**b).[2]

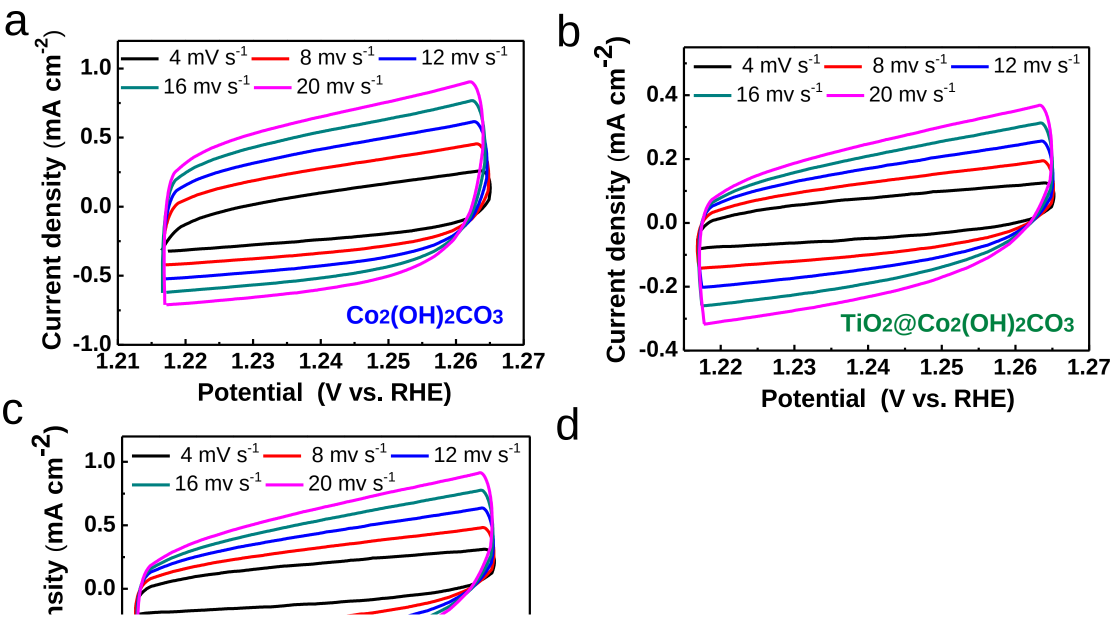

Figure S10. CV curves of different electrodes in double layer region at scan rates of 4, 8, 12, 16 and 20 mV s^{-1}, respectively.: (a) $Co_2(OH)_2CO_3$; (b) TiO_2@$Co(OH)_2CO_3$; (c)Co_9S_8; (d) TiO_2@Co_9S_8 electrodes.

Table S1 Electrocatalytic comparison for different catalysts

Catalyst	OER Overpotential (mV vs. RHE)	Tafel slope (mV Dec^{-1})	HER Overpotential (mV vs. RHE)	Tafel slope (mV Dec^{-1})	Ref.

$Co_2(OH)_2CO_3$	330	79	197	102	This work
$TiO_2@Co_2(OH)_2O_3$	350	89	226	126	This work
Co_9S_8	276	73	222	85	This work
$TiO_2@Co_9S_8$	240	55	139	65	This work
$S\text{-}CuCo_2O_4$	/	/	154	180	[3]
$Co_9S_8@C$	/	/	280	/	[4]
cobalt-sulfide	/	/	160	93	[5]
Co_2P nanorods	/	/	155	71	[6]
NiMo@N-C	/	/	130	84	[7]
NiFe@N-C	297	48	/	/	[7]
Co-P	345	42	94	47	[8]
Co_9S_8/graphene	409	82	/	/	[9]
Co_9S_8@N, S-C	310	68	/	/	[10]
Ni_3S_2 nanorods	157	159	/	/	[11]
NiS	290	89	140	83	[12]
NiFeS–Fe/NF	101	117	/	/	[13]
Ni_2P	290	/	/	/	[14]

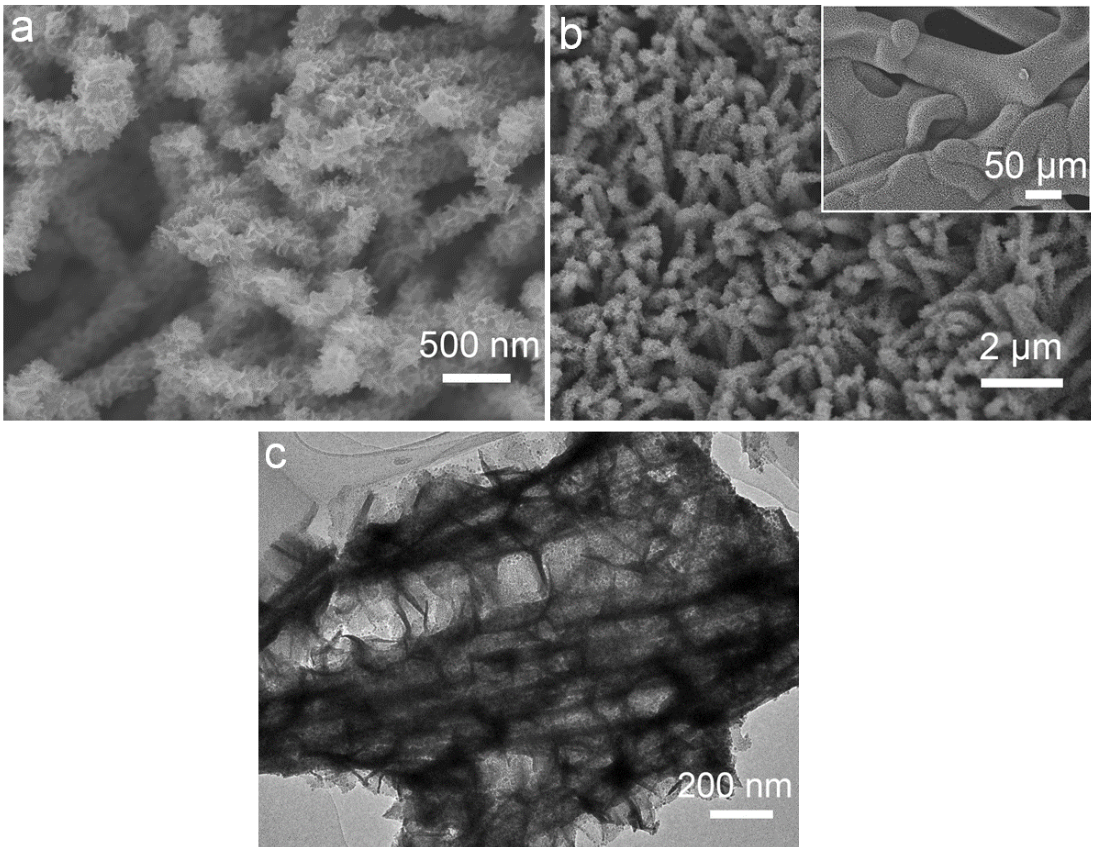

Figure S11. SEM images of $TiO_2@Co_9S_8$ electrode after 30 h test at 10 mA cm^{-2} during overall water splitting.

References

[1] X. Xia, Z. Zeng, X. Li, Y. Zhang, J. Tu, N. C. Fan, H. Zhang, H. J. Fan, *Nanoscale* **2013**, *5*, 6040.

[2] Y. Cong, J. Zhang, F. Chen, M. Anpo, *J. Phys. Chem. C* **2007**, *111*, 6976.

[3] Y. Gong, Y. Zhao, Y. Chen, Y. Wang, C. Sun, *RSC Adv.* **2016**, *6*, 43185.

[4] L.-L. Feng, G.-D. Li, Y. Liu, Y. Wu, H. Chen, Y. Wang, Y.-C. Zou, D. Wang, X. Zou, *ACS Appl. Mater. Interfaces* **2015**, *7*, 980.

[5] Y. Sun, C. Liu, D. C. Grauer, J. Yano, J. R. Long, P. Yang, C. J. Chang, *J. Am. Chem. Soc.* **2013**, *135*, 17699.

[6] Z. Huang, Z. Chen, Z. Chen, C. Lv, M. G. Humphrey, C. Zhang, *Nano Energy* **2014**, *9*, 373.

[7] Y. Zhang, X. Xia, X. Cao, B. Zhang, N. H. Tiep, H. He, S. Chen, Y. Huang, H. J. Fan, *Adv. Energy Mater.* **2017**.

[8] N. Jiang, B. You, M. Sheng, Y. Sun, *Angewandte Chemie* **2015**, *127*, 6349.

[9] S. Dou, L. Tao, J. Huo, S. Wang, L. Dai, *Energy Environ. Sci.* **2016**, *9*, 1320.

[10] H.-x. Zhong, K. Li, Q. Zhang, J. Wang, F.-l. Meng, Z.-j. Wu, J.-m. Yan, X.-b. Zhang, *NPG Asia Mater.* **2016**, *8*, e308.

[11] W. Zhou, X.-J. Wu, X. Cao, X. Huang, C. Tan, J. Tian, H. Liu, J. Wang, H. Zhang, *Energy Environ. Sci.* **2013**, *6*, 2921.

[12] W. Zhu, X. Yue, W. Zhang, S. Yu, Y. Zhang, J. Wang, J. Wang, *Chem. Commun.* **2016**, *52*, 1486.

[13] B. Dong, X. Zhao, G.-Q. Han, X. Li, X. Shang, Y.-R. Liu, W.-H. Hu, Y.-M. Chai, H. Zhao, C.-G. Liu, *J. Mater. Chem. A* **2016**, *4*, 13499.

[14] L.-A. Stern, L. Feng, F. Song, X. Hu, *Energy Environ. Sci.* **2015**, *8*, 2347.
